# Updated Information on Antimicrobial Activity of Hydrazide–Hydrazones

**DOI:** 10.3390/ijms22179389

**Published:** 2021-08-30

**Authors:** Łukasz Popiołek

**Affiliations:** Chair and Department of Organic Chemistry, Faculty of Pharmacy, Medical University of Lublin, 4A Chodźki Street, 20-093 Lublin, Poland; lukasz.popiolek@umlub.pl; Tel.: +48-81-448-72-43

**Keywords:** hydrazide–hydrazone, biological activity, antibacterial activity, antitubercular activity, antifungal activity

## Abstract

Hydrazide–hydrazones possess a wide spectrum of bioactivity, including antibacterial, antitubercular, antifungal, anticancer, anti-inflammatory, anticonvulsant, antidepressant, antiviral, and antiprotozoal properties. This review is focused on the latest scientific reports regarding antibacterial, antimycobacterial, and antifungal activities of hydrazide–hydrazones published between 2017 and 2021. The molecules and their chemical structures presented in this article are the most active derivatives, with discussed activities having a hydrazide–hydrazone moiety as the main scaffold or as a side chain. Presented information constitute a concise summary, which may be used as a practical guide for further design of new molecules with antimicrobial activity.

## 1. Introduction

In the field of medicinal chemistry, hydrazide–hydrazones are still in continuous interest due to their diverse and wide spectrum of biological properties [1,2,3,4]. Additionally, hydrazide–hydrazones are versatile compounds for the synthesis of heterocyclic systems [1,3,5,6], preparing metal complexes and are used as ligands in coordination chemistry [7,8,9,10].

Among bioactivity profiles of hydrazide–hydrazones, antimicrobial properties are the most common in the scientific literature [11,12,13,14,15,16,17,18,19,20,21,22,23,24,25,26,27,28,29,30,31,32,33,34,35,36,37,38,39,40,41,42,43,44,45,46]. This is especially important due to the fact that bacterial and fungal infections became more and more difficult and sometimes impossible to treat as a result of the increase of antibiotic and chemotherapeutic resistant strains [47]. It is worth mentioning that hydrazide–hydrazone moiety is also present in the chemical structure of medicines with antimicrobial activity, such as nitrofurazone, furazolidone, or nitrofurantoin [3].

This review is an update and continuation of the review, which was previously published in 2017 [3], and focuses on the most recently described (2017–2021) potent hydrazide–hydrazones with applications as antibacterial, antimycobacterial, and antifungal agents.

## 2. Antimicrobial Activity

### 2.1. Antibacterial Activity

The most frequently encountered in the scientific literature are hydrazide–hydrazones which possess antibacterial activity. Searching for such compounds among this group seems reasonable due to the fact that many derivatives of this class are highly active even against resistant strains; this is especially important nowadays when many bacteria become resistant to commonly used medicines [11,12,13,14,15,16,17,18,19,20,21,22,23,24,25,26,27,28,29,30,31,32,33,34,35,36,37,38,39,40,41,42].

**Figure 1:**
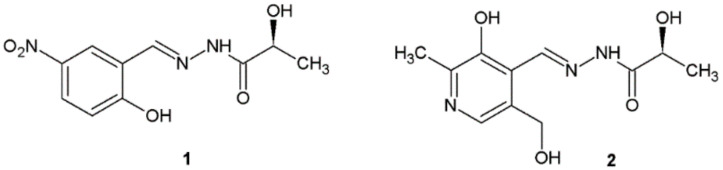
New hydrazide–hydrazones of lactic acid with antibacterial activity.

Noshiranzadeh et al. conducted the synthesis of a series of new hydrazide–hydrazones of lactic acid and evaluated their antibacterial activity against four bacterial strains (*S. aureus*, *S. pneumoniae*, *E. coli*, and *P. aeruginosa*) using the broth microdilution method. Compounds **1** and **2** showed higher antibacterial activity (Minimal inhibitory concentration MIC = 64–128 µg/mL) than the other compounds but lower than gentamicin used as a reference substance (Figure 1). The high antibacterial activity of compound **1** is possibly attributed to the presence of an electronegative NO_2_ substituent. The authors concluded that the compounds with electron-withdrawing groups like I, Br, or NO_2_ generally showed better antibacterial activity in comparison with the compounds containing electron-donating OCH_3_ or OH groups [11].

Heidari et al. published the study which aimed to investigate the effects of sub-minimum inhibitory concentrations of compound **1** against *Pseudomonas aeruginosa* PAO1 quorum sensing related virulence factors (Figure 1). Treated PAO1 cultures in the presence of this hydrazide–hydrazone at subinhibitory concentrations showed significant inhibition of virulence factors, including motility, biofilm formation, alginate and pyocyanin production, and susceptibility to H_2_O_2_ (*p* <0.001). The authors suggested that such action may be the mechanism of activity of this compound against *Pseudomonas aeruginosa* PAO1 [12].

**Figure 2:**
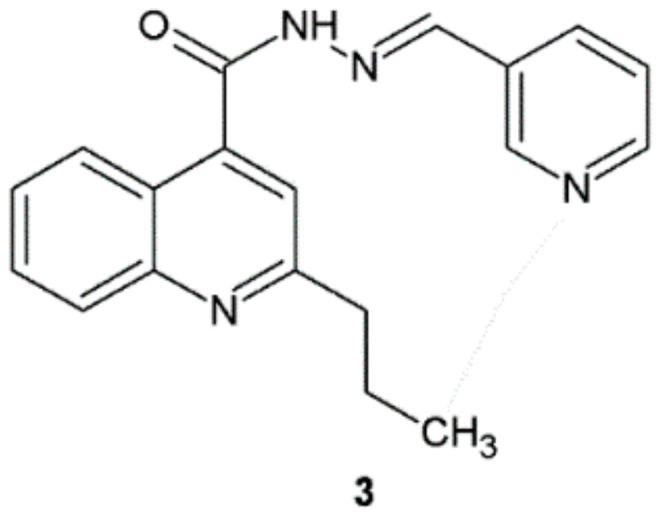
Quinoline derivative with significant antibacterial properties.

Olayinka et al. synthesized a series of new hydrazide–hydrazones of 2-propylquinoline-4-carboxylic acid and carried out antibacterial activity screening towards six bacterial strains (*P. aeruginosa*, *S. aureus*, *E. coli*, *Proteus vulgaris*, *Bacillus licheniformis*, and *Micrococcus varians*) using the agar diffusion method. Compound **3** was the hydrazide–hydrazone with the lowest MIC value in the range of 0.39 ± 0.02–1.56 ± 0.02 µg/mL across all the microorganisms screened (Table 1, Figure 2). Authors proved that the presence of electron-donating group (EDG) at position 4 and electron-withdrawing group (EWG) at position 2 in the phenyl ring had a crucial effect on the antibacterial activity [13].

**Figure 3:**
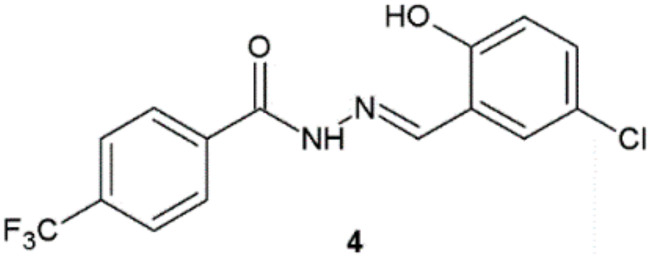
Hydrazide–hydrazone of 4-trifluoromethylbenzoic acid with high antibacterial activity.

Krátký et al. synthesized a series of new hydrazide–hydrazones of 4-trifluoromethylbenzoic acid and evaluated them as possible antibacterial agents. The majority of obtained hydrazide–hydrazones were only slightly active. The highest activity superior to bacitracin (BAC), used as a reference substance, was shown by compound **4** (Table 2, Figure 3). This substance did not show cytotoxicity towards HepG2 cells (hepatocellular carcinoma cells) and BMMΦ (murine bone marrow culture-derived macrophages) (IC_50_ >100 µM) [14].

**Figure 4:**
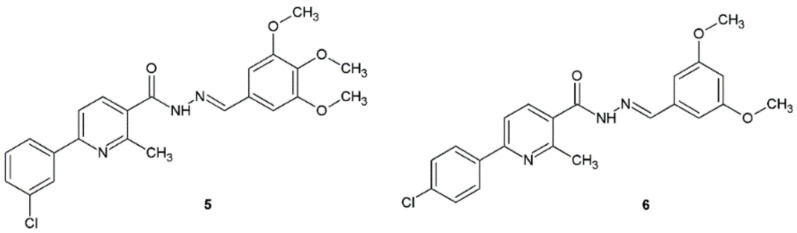
Hydrazide–hydrazones active against *S. pneumoniae* and *E. coli*.

Abdelrahman et al. synthesized novel hydrazide–hydrazones and evaluated in vitro their antibacterial properties against two Gram-positive bacteria: *Streptococcus pneumoniae* RCMB 010010, *S. aureus* RCMB 010028 and two Gram-negative bacteria: *P. aeruginosa* RCMB 010043, *E. coli* RCMB 010052. Compounds **5** and **6** displayed significant and higher antibacterial activity when compared with ampicillin and ciprofloxacin, respectively (Figure 4). Compounds **5** and **6** showed two-fold increased inhibition against *S. pneumoniae* with MIC = 0.49 µg/mL, compared to ampicillin (MIC = 0.98 µg/mL). The authors proved that compounds bearing electron-donating groups showed better activities than the electron-withdrawing ones. Regarding Gram-negative bacteria, remarkable activity was elicited by the derivatives **5** and **6** against *E. coli* (MIC = 0.49 µg/mL), showing two-fold the potency of the standard ciprofloxacin (MIC = 0.98 µg/mL). The authors tested synthesized compounds for cytotoxic activities against human lung fibroblast normal cells (WI-38). Hydrazide–hydrazones **5** and **6** showed no cytotoxic activity at 0–500 µg/mL concentrations [15].

**Figure 5:**
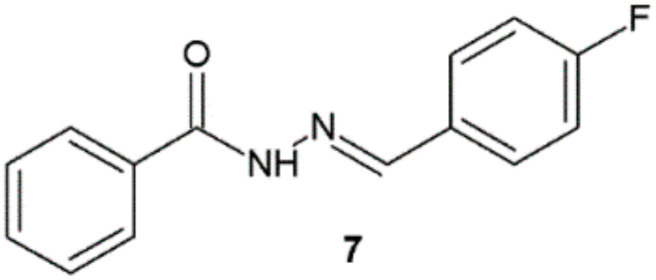
Hydrazide–hydrazone **7** with antibacterial properties.

Analysis of the values of the zone of inhibition growth of compounds synthesized by Manikandan et al. revealed that among obtained hydrazide–hydrazones, only derivative with 4-fluorophenyl substituent **7** had shown satisfactory antibacterial sensitivity in comparison with ciprofloxacin (Figure 5, Table 3) [16].

**Figure 6:**
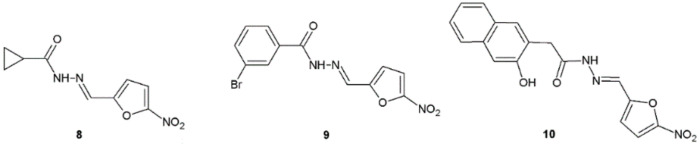
Nitrofurazone analogues with significant antibacterial activity.

The results of the study by Popiołek and Biernasiuk indicated that synthesized and in vitro examined hydrazide–hydrazones exhibited a wide spectrum of antibacterial activity against tested reference bacteria. Substances **8**, **9**, and **10** were especially potent (MIC = 0.002–0.98 µg/mL) against Gram-positive bacteria (Figure 6). *Staphylococcus epidermidis* ATCC 12228 was the most sensitive to all synthesized compounds, while *Micrococcus luteus* ATCC 10240 was the least susceptible. Compounds **8** and **9** showed almost two thousand times higher activity (MIC < 1 µg/mL) than nitrofurantoin (MIC = 3.91 µg/mL) against *B. subtilis* ATCC 6633 and *S. epidermidis* ATCC 12228, respectively. The MIC value of compound **9** (MIC = 0.002 µg/mL) was sixty-one times lower than the MIC of ciprofloxacin (MIC = 0.122 µg/mL) against *S. epidermidis* ATCC 12228. Compound **8** had MIC value (MIC = 0.002 µg/mL) against *B. subtilis* ATCC 6633, which was almost eight thousand times lower than the MIC of cefuroxime (MIC = 15.63 µg/mL) [17].

**Figure 7:**
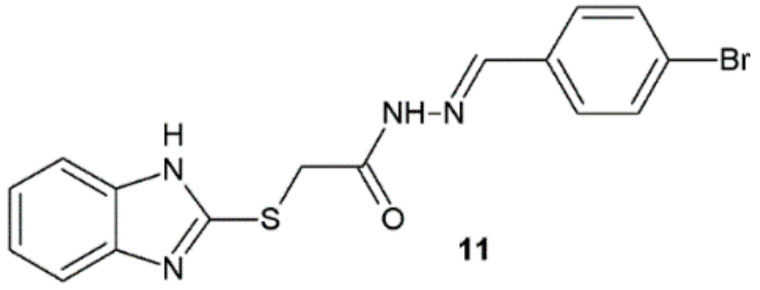
Benzimidazole derivative active against bacterial strains.

Yadav et al. synthesized novel hydrazide–hydrazones as a derivative of benzimidazole and evaluated their potency against bacterial strains. Among obtained molecules, compound **11** displayed far better antibacterial activity against *S. aureus*, *B. subtilis*, and *E. coli* (MIC = 0.032 µM) in comparison with cefadroxil used as a reference substance (MIC = 0.345 µM) (Figure 7) [18].

**Figure 8:**
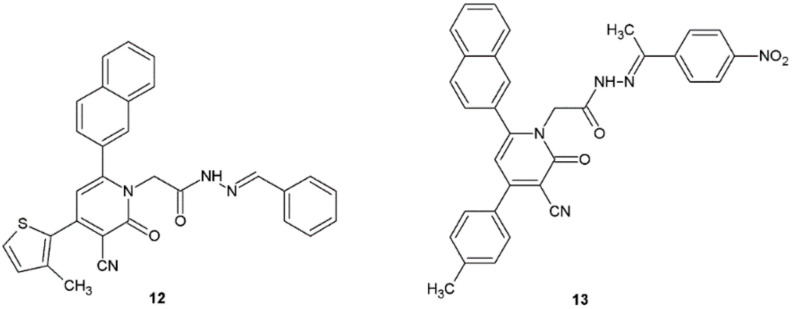
2-Oxonicotinonitrile derivatives with antibacterial properties.

Hydrazide–hydrazones obtained by El-Sayed et al. showed good or moderate activity against Gram-positive bacteria: *B. subtilis*, *B. cereus*, and Gram-negative bacteria: *P. aeruginosa, E. coli*. Compounds **12** and **13** displayed higher activity against Gram-positive *B. subtilis* in comparison with cefotaxime used as positive control (Figure 8, Table 4) [19].

**Figure 9:**
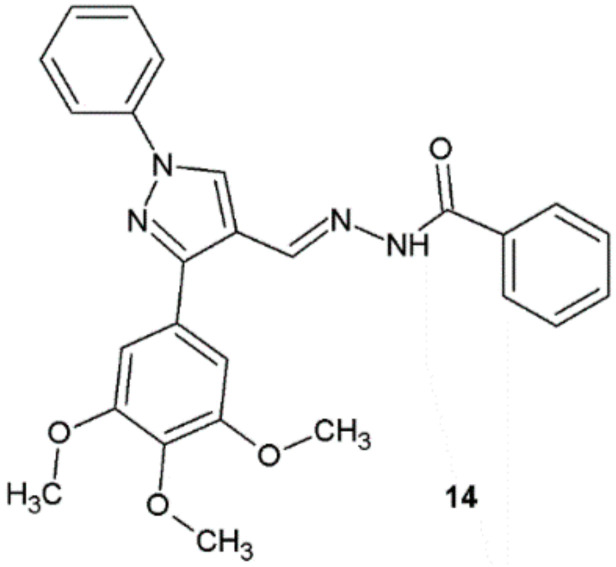
Pyrazole derivative with antibacterial properties.

Hydrazide–hydrazones synthesized by Chennapragada et al. were screened against *E. coli* MTCC 443, *P. aeruginosa* MTCC 2453, *S. aureus* MTCC 3160, and *B. cereus* MTCC 1305. Antibacterial activity was assessed on the basis of the measurement of the diameter of the zone of inhibition growth (ZOI). Compound **14** exhibited significant antibacterial activity at all concentrations in comparison with streptomycin used as positive control (Figure 9) [20].

**Figure 10:**
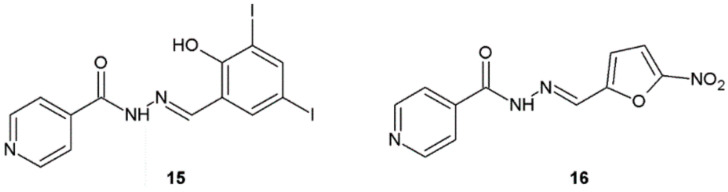
Hydrazide–hydrazones of isonicotinic acid with significant antibacterial activity.

In 2018, Popiołek et al. published an article in which they described the synthesis and antibacterial activity analysis of new hydrazide–hydrazones of isonicotinic acid. The most significant activity among obtained compounds was displayed by substances **15** and **16**. Hydrazide–hydrazone **15** exhibited very strong activity towards all tested Gram-positive bacteria (MIC = 1.95–7.81 μg/mL, MBC = 3.91–125 μg/mL). This substance showed bactericidal action against *S. aureus* ATCC 6538, *S. epidermidis* ATCC 12228, and *B. subtilis* ATCC 6633 (MBC/MIC = 2–4) and bacteriostatic effect against other bacteria (MBC/MIC = 8–32). The compound **16** also showed very strong activity towards Gram-positive bacteria (MIC = MBC = 3.91–7.81 μg/mL, MBC/MIC = 1–2). Its activity against *S. aureus* ATCC 25923 and *S. aureus* ATCC 6538 (MIC = 3.91 μg/mL) was four times higher than the activity of nitrofurantoin (MIC = 15.62 μg/mL) with bactericidal effect (Figure 10) [21].

**Figure 11:**
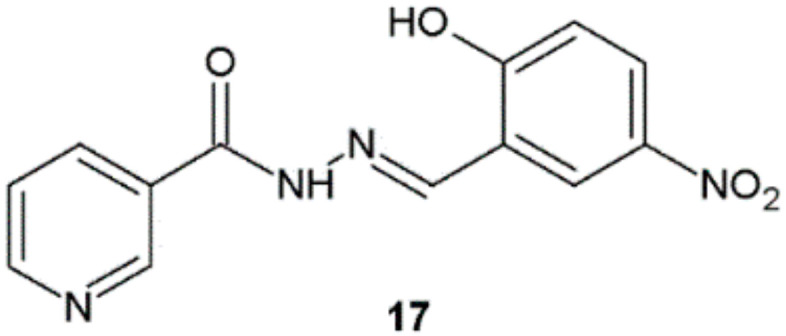
Hydrazide–hydrazone of nicotinic acid with good inhibition of bacterial growth.

Similarly, Polović et al., in 2019, synthesized novel series of hydrazide–hydrazones of nicotinic acid and assessed their antibacterial activity. Antibacterial assays performed in this study showed that compound **17** with nitro group displayed good inhibition of bacterial growth, whereas the compounds without an electron-withdrawing group (chloro- and nitro-) showed weak antibacterial activity (Figure 11, Table 5). According to the authors, this may be due to the fact that electron-withdrawing substituents increase the lipophilicity of the compounds, which leads to higher partitioning of such compounds into the lipophilic phase of a microbial membrane [22].

**Figure 12:**
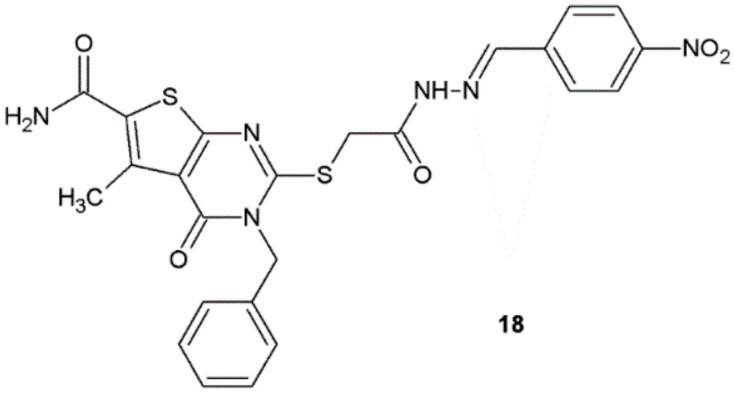
Pyrimidine derivative with antibacterial properties.

All the newly synthesized hydrazide–hydrazones by Shaaban et al. were evaluated for their in vitro antibacterial activity against *S. aureus* RCMB 0100183, *B. subtilis* RCMB 0100162, *S. epidermidis* RCMB 0100183, *P. aeruginosa* RCMB 0100243, *P. vulgaris* RCMB 010085, and *E. coli* RCMB 010052. Ampicillin and levofloxacin were used as reference standard antibacterial agents. Compound **18** exhibited moderate activity against Gram-positive and Gram-negative bacteria (Table 6, Figure 12) [23].

**Figure 13:**
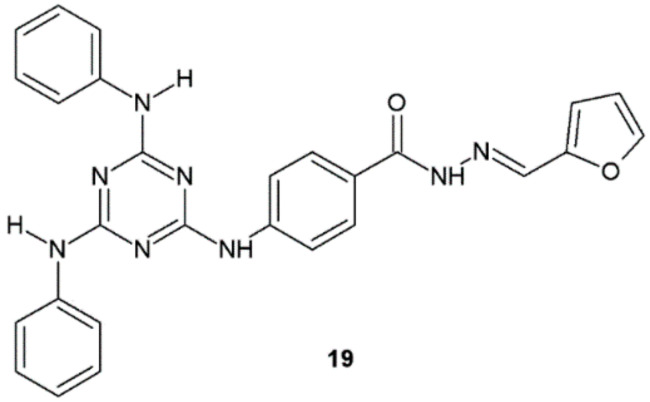
*s*-Triazine derivative active against *E. coli* and *S. aureus*.

Hydrazide–hydrazones obtained by Haiba et al. exhibited promising antibacterial activity against bacterial strains. Compound **19** showed two-fold higher antibacterial activity against *E. coli* (MIC = 12.5 μg/mL) and *S. aureus* (MIC = 6.25 μg/mL) than ampicillin (MIC = 25 and 12.5 μg/mL, respectively) (Figure 13). Additionally, compound **19** also showed significant antibacterial activity against MDR clinical isolates of *K. pneumoniae* (MIC = 12.5 μg/mL) and methicillin-resistant *S. aureus* MRSA1 (MIC = 3.125 μg/mL). The cytotoxicity of hydrazide–hydrazone **19** was tested in a VERO (African green monkey kidney) cell line. The performed test revealed that 50% cytotoxic concentration value CC_50_ equals 125, which, according to the authors, corresponds to good safety profile of this substance. The authors also performed a molecular docking study to find potential mechanism of antibacterial action of synthesized compounds. As a result, it occurred that the antibacterial potency of obtained hydrazide–hydrazones may be connected with strong binding interactions in the DNA gyrase active site [24].

**Figure 14:**
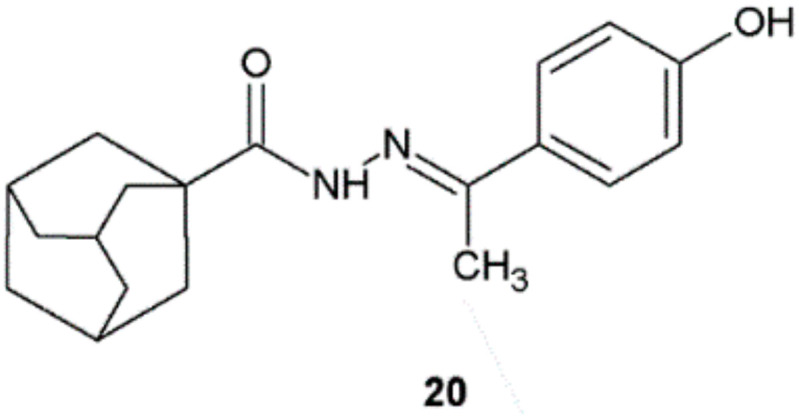
Adamantane derivative with antibacterial activity.

The results of antimicrobial activity testing of compounds synthesized by Phan et al. showed that all newly synthesized hydrazide–hydrazones possessed various inhibition effects on the tested Gram-positive bacteria. Strong antibacterial activity was found for compound **20** in comparison with streptomycin (Figure 14, Table 7) [25].

**Figure 15:**
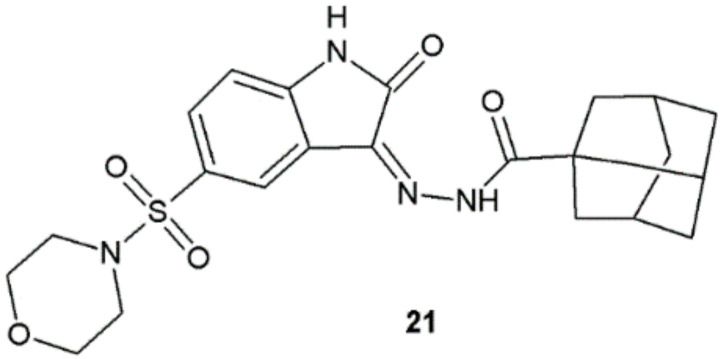
Indol-2-one derivative with antibacterial activity.

The most active compound among indol-2-one derivatives synthesized by Salem et al. was **21**. It showed higher antibacterial activity than tetracycline against *B. subtilis*, *S. aureus*, and *E. coli* (Figure 15, Table 8). In order to determine the possible mechanism of action of synthesized compounds, the authors performed the inhibitory activity assay against DNA gyrase isolated from *S. aureus*. Hydrazide–hydrazone **21** showed strong inhibition of gyrase (IC_50_ = 19.32 ± 0.99 µM) when compared to ciprofloxacin (IC_50_ = 26.43 ± 0.64 µM) [26].

**Figure 16:**
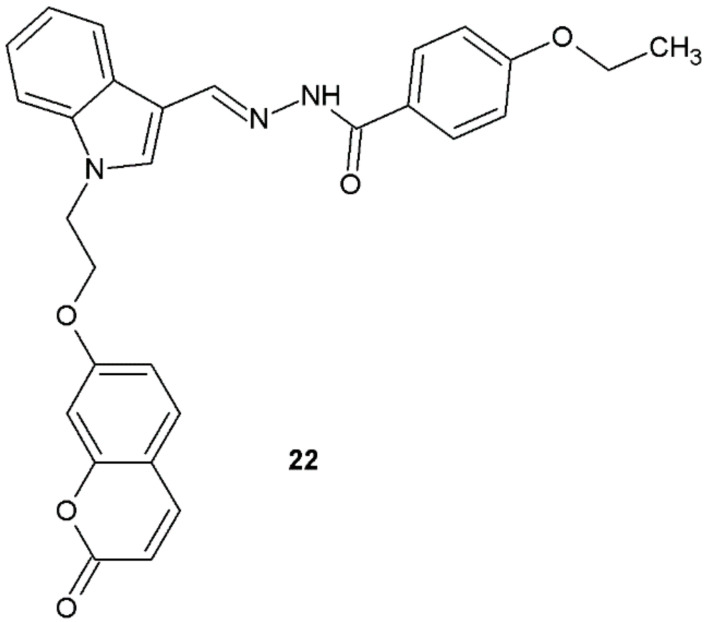
*N*-substituted indole derivative with antibacterial properties.

The results of antibacterial activity screening of hydrazide–hydrazones synthesized by Tiwari et al. indicated that the compounds were active against Gram-positive bacteria. The highest activity was shown by compound **22** (Figure 16, Table 9) [27].

**Figure 17:**
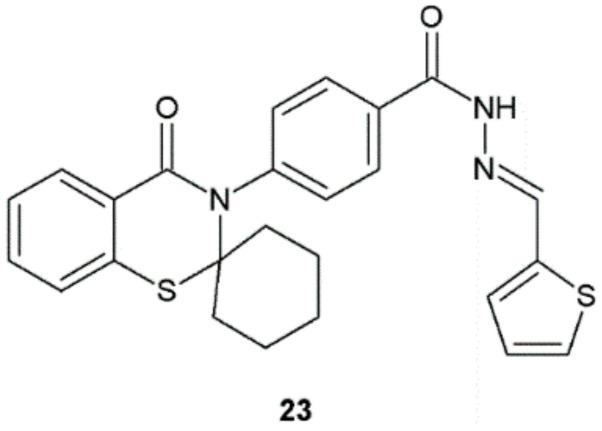
Benzo[e][1,3]thiazine derivative with promising antibacterial properties.

The hydrazide–hydrazone **23** obtained by Ewies et al. exhibited promising antibacterial activity against *S. aureus*, *S. typhimurium*, and *P. aeruginosa* (Figure 17, Table 10) [28].

**Figure 18:**
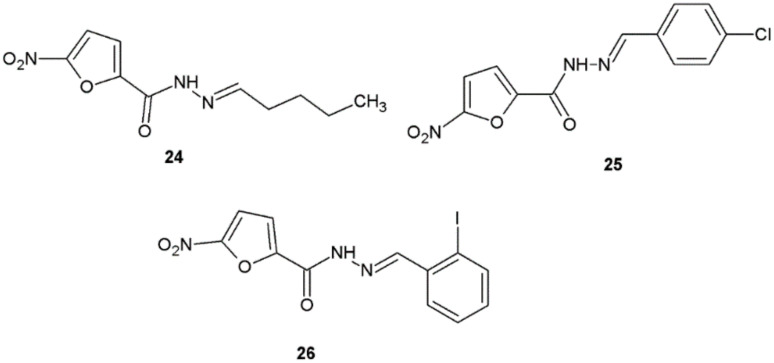
Novel derivatives of 5-nitrofuran-2-carboxylic acid with antibacterial activity.

In 2020, Popiołek et al. reported the synthesis and antimicrobial activity potential of hydrazide–hydrazones of 5-nitrofuran-2-carboxylic acid. Majority of obtained substances showed high antibacterial activity with bactericidal effect against tested microorganisms (MIC = 0.48–15.62 μg/mL, MBC = 0.98–62.5 μg/mL, MBC/MIC = 1–4). The most sensitive for tested hydrazide–hydrazones were *S. epidermidis* ATCC 12228, *S. aureus* ATCC 43300, *S. aureus* ATCC 6538, *B. subtilis* ATCC 6633, and *B. cereus* ATCC 10876. The highest antibacterial activity among synthesized derivatives was shown by compounds **24**, **25**, and **26** (Figure 18) [29].

**Figure 19:**
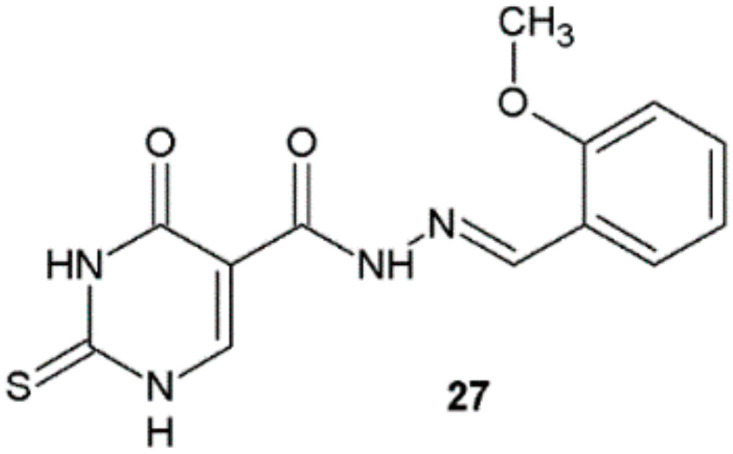
*N*-(2-Thiouracil-5-oyl)hydrazone derivative with antibacterial activity.

In 2021, El-Etrawy et al. synthesized novel series of 2-thiouracil derivatives and evaluated their in vitro antibacterial properties. Compound **27** was found to possess the highest antibacterial activity on the basis of the measurement of the zone of inhibition growth (Figure 19, Table 11) [30].

**Figure 20:**
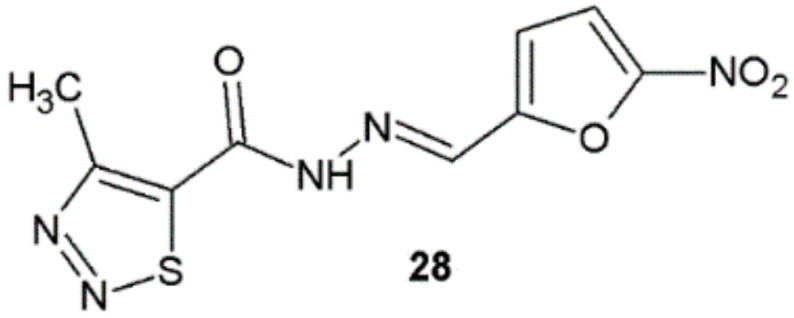
4-Methyl-1,2,3-thiadiazole-carboxylic acid hydrazide derivative active against a panel of bacterial strains.

Novel derivatives of 1,2,3-thiadiazole synthesized by Paruch et al. displayed interesting antibacterial properties. Hydrazide–hydrazone **28** showed a bactericidal effect among almost all tested strains (Figure 20). The MIC values of this substance, which inhibited bacterial growth, ranged from 1.95 μg/mL (for *Staphylococcus* spp.) to 15.62 μg/mL (for *E. faecalis* ATCC 29212). The activity of this derivative against *S. aureus* ATCC 25923 and ATCC 43300 was two-fold greater than for nitrofurantoin. Towards *S. aureus* ATCC 6538 strain was seven times higher than for nitrofurantoin. This compound also possessed good activity against *S. epidermidis* ATCC 12228 and *M. luteus* ATCC 10240, two and eight times higher, respectively, in comparison with nitrofurantoin [31].

### 2.2. Antimycobacterial Activity

On the basis of the survey of scientific literature, it can be concluded that hydrazide–hydrazones may also be regarded as promising antitubercular agents, which is especially important when tuberculosis is still a serious threat for people [32].

The study performed by Abdelrahman et al. revealed that compound **6** also possessed superior activity against *M. tuberculosis* with MIC = 0.39 µg/mL, two-fold higher activity than that of isoniazid (MIC = 0.75 µg/mL) (Figure 4) [15].

**Figure 21:**
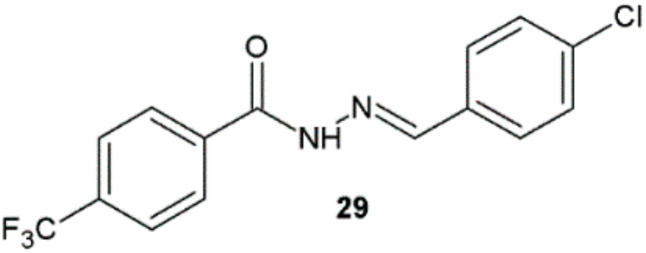
Hydrazide–hydrazone of 4-trifluoromethylbenzoic acid with antimycobacterial activity.

Among hydrazide–hydrazones of 4-trifluoromethylbenzoic acid synthesized by Krátký et al., compound **29** showed high antitubercular activity comparable to isoniazid against the clinical isolate of *Mycobacterium kansasii* 6509/96 (Figure 21, Table 12) [14].

**Figure 22:**
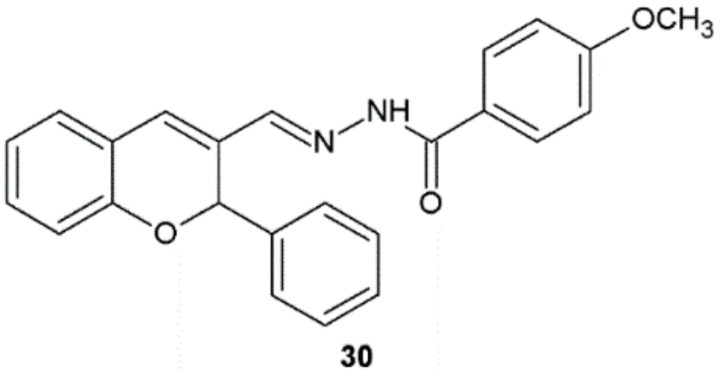
2*H*-Chromene derivative active against *M. tuberculosis* H37Rv.

In the study performed by Angelova et al., synthesized hydrazide–hydrazones with 2*H*-chromene and coumarin scaffold were evaluated in vitro against *M. tuberculosis* H37Rv. Compound **30** proved to be the most active against tested strain (MIC = 0.13 µM) (Figure 22). Isoniazid and ethambutol showed higher values of MIC 1.45 and 7.64 µM, respectively. Hydrazide–hydrazone **30** showed low cytotoxicity against human embryonal kidney cell line HEK-293T (IC_50_ = 90.66 µM) and displayed a favorable selectivity index (SI = 697.38). Authors suggested that this class of hydrazide–hydrazones may be regarded as a promising new candidate for further investigations as an antitubercular agent [33].

**Figure 23:**
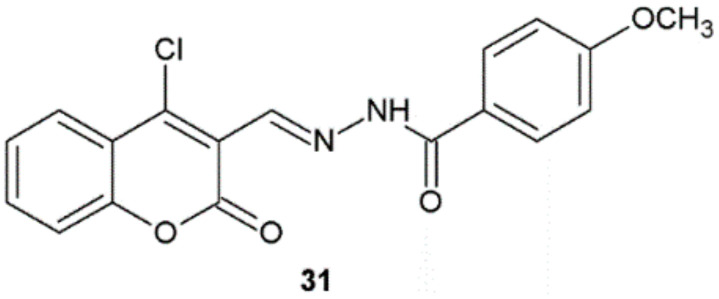
Hydrazide–hydrazone with significant antitubercular activity.

In 2017, Angelova et al. published a similar study that concerned the in vitro antimycobacterial activity of benzopyran derivatives against the reference strain of *M. tuberculosis* H37Rv. The most active was compound **31** (MIC = 0.28 µM) with *p*-methoxyphenyl substituent (Figure 23). Its activity was higher than that of isoniazid (MIC = 0.79 µM) and ethambutol (MIC = 1.46 µM). The authors observed that the presence of OH, OCH_3_, and N(CH)_3_ functional groups at four position in the phenyl ring enhanced the antimycobacterial activity. This compound was also tested for cytotoxicity against the human embryonic kidney cell line HEK-293T. According to the authors, it showed minimal cytotoxicity (IC_50_ = 112.9 µM) and a high value of selectivity index (SI = 403). In order to discover the potential mechanism of antimycobacterial activity of synthesized compounds, the authors performed molecular docking studies and investigated binding to the 2-trans-enoyl-ACP reductase (InhA) enzyme involved in *M. tuberculosis* cell wall biogenesis. On the basis of obtained results, the authors suggested that the activity of synthesized molecules may be connected with interactions with the inhibitor binding cavity of *M. tuberculosis* enoyl-ACP reductase and/or related to the ability of the tested compounds to penetrate mycobacterial cells [34].

**Figure 24:**
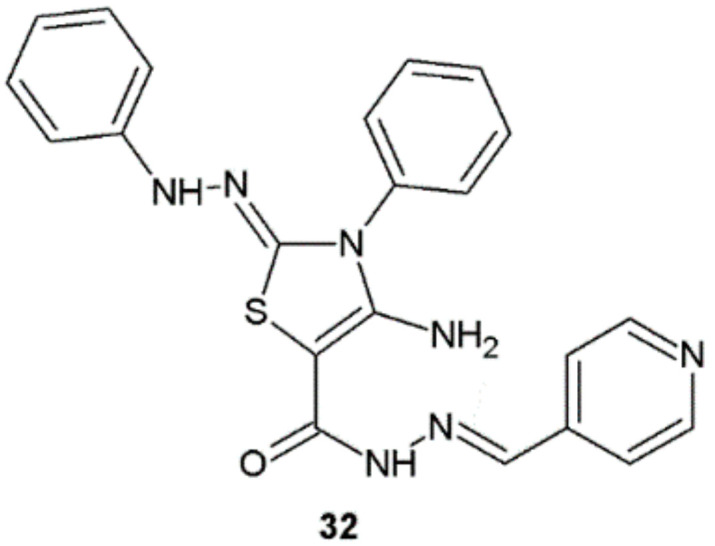
Chemical structure of compound **32** with antimycobacterial activity.

Hydrazide–hydrazones synthesized and tested for potential antimycobacterial activity by Atta et al. displayed high potency against *M. tuberculosis* H37Rv. Compound **32** showed the highest activity with MIC = 7.30 μM and was equipotent to ethambutol (MIC = 7.64 μM) and seven times more active than pyrazinamide (MIC = 50.77 μM), which were used as reference compounds (Figure 24). Substance **32** was also examined for cytotoxicity in human embryonic kidney (HEK 293) cell line at the concentration of 50 μg/mL with the use of MTT assay. It showed a lower cytotoxic effect (20.08% inhibition) than standard antimycobacterial medicine—isoniazid (35.60% inhibition) [35].

**Figure 25:**
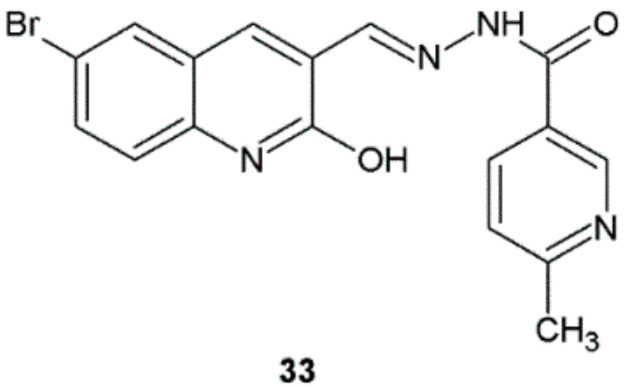
Quinoline derivative with antimycobacterial properties.

Among hydrazide–hydrazones obtained by Mandewale et al., compound **33** showed the highest antitubercular activity against *M. tuberculosis* H37Rv ATCC 27294 (MIC = 32.55 μM) but lower than reference substance pyrazinamide (MIC = 25.34 μM) (Figure 25) [36].

**Figure 26:**
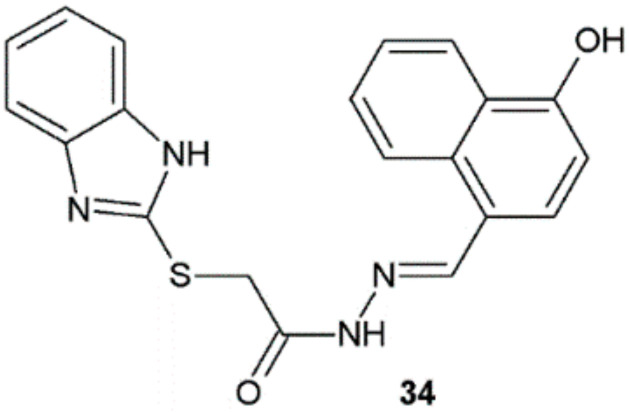
Benzimidazole derivative active against *M. tuberculosis* H37Rv.

Derivatives of benzimidazole synthesized by Yadav et al. were also evaluated for potential antimycobacterial activity. The activity equal to the activity of streptomycin was shown by compound **34** (MIC = 12.5 µg/mL) against *M. tuberculosis* H37Rv (Figure 26) [18].

**Figure 27:**
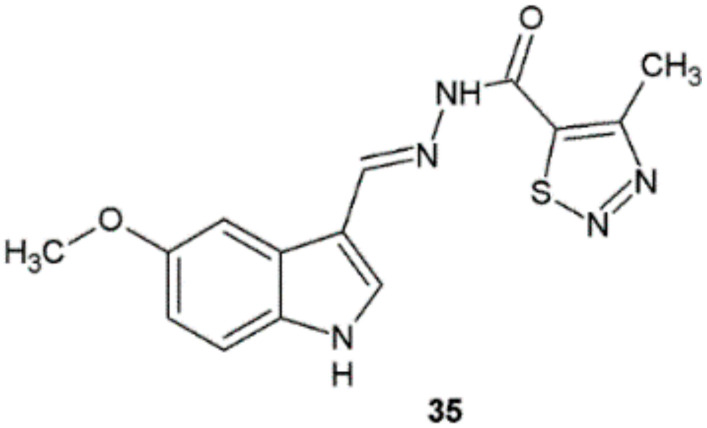
Novel hydrazide–hydrazone with antitubercular properties.

The structure–activity relationship study of indole derivatives synthesized by Angelova et al. with respect to their antitubercular activity revealed that compounds with 5-methoxysubstituted indole scaffold were found to be the most potent molecules with MIC values in the 0.39–0.77 μM range against *M. tuberculosis* H37Rv. Among the tested compounds, the highest antimycobacterial activity, selectivity (SI > 1978.83), and low toxicity were found for compound **35** (MIC = 0.3969 μM). Ethambutol and isoniazid showed higher values in this study (MIC = 1.6996 and 0.9115 μM, respectively) (Figure 27) [37].

**Figure 28:**
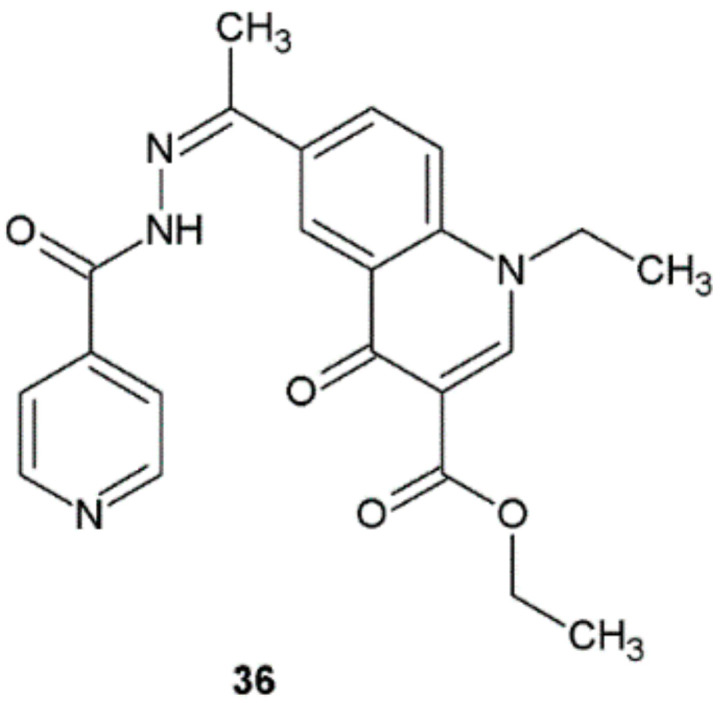
Quinolone–isoniazid hybrid active against *M. tuberculosis* H37Rv.

In 2019, Beteck et al. published an article that concerned the synthesis and antitubercular activity against *M. tuberculosis* H37Rv of quinolone–isoniazid hybrids. The antimycobacterial activities were reported as minimal inhibitory concentration (MIC_90_) required to inhibit 90% of mycobacterial growth. All of the obtained compounds exhibited antimycobacterial activity. Many of them had MIC_90_ values less than 3 μM. The highest activity was shown by compound **36** (MIC = 0.2 μM) (Figure 28). The activity of this compound was equal to the activity of isoniazid [38].

**Figure 29:**
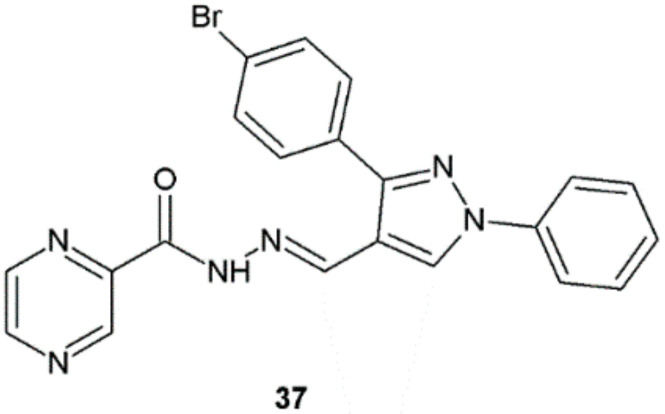
Pyrazine derivative with antitubercular properties.

Hassan et al. synthesized novel pyrazine derivatives with hydrazide–hydrazone moiety, which appeared to be effective inhibitors of the growth of *M. tuberculosis* H37Rv ATCC 27294. The highest potency was shown by compound **37**, which displayed significant anti-TB activity with MIC value of 0.78 μg/mL, which equals two times the activity of ethambutol (MIC = 1.56 μg/mL) and eight times the activity of pyrazinamide (MIC = 6.25 μg/mL) (Figure 29). In the in vitro cytotoxicity assay against PBMC (peripheral blood mononuclear cells) normal cell line, this hydrazide–hydrazone showed no cytotoxicity (IC_50_ = 846.9 µg/mL) and very high selectivity index (SI = 1085.7). To find a possible mechanism of activity of obtained hydrazide–hydrazones, the authors processed a docking study into the active site of the pantothenate synthetase enzyme. Substance **37** revealed to have favorable binding modes and interaction patterns with the active site of the enzyme [39].

**Figure 30:**
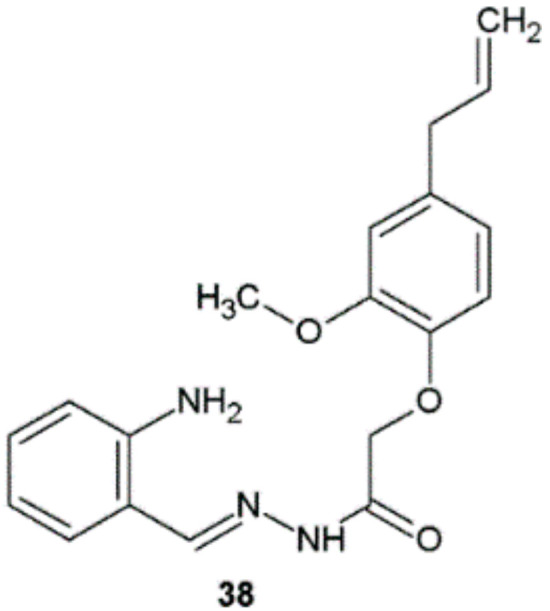
Eugenol derivative with antimycobacterial potential.

Novel hydrazide–hydrazones synthesized from eugenol were tested for antimycobacterial potential. Among synthesized derivatives, compound **38** possessed the highest sensitivity against *M. tuberculosis* H37Rv at 25 µg/mL level (Figure 30) [40].

**Figure 31:**
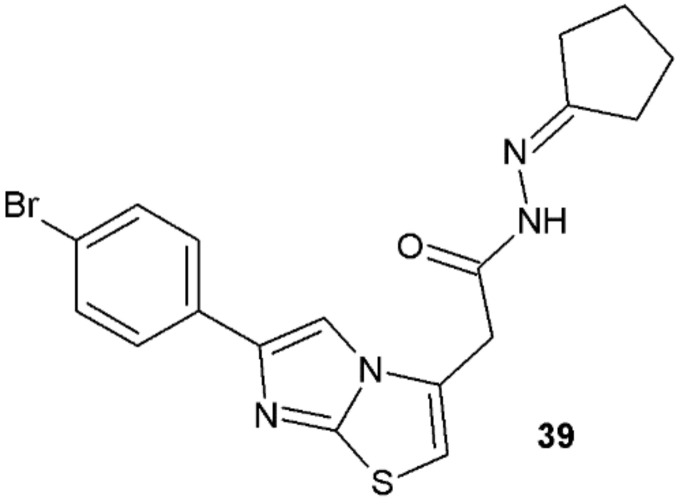
Hydrazide–hydrazone with antimycobacterial activity.

Gürsoy et al., in 2020, obtained novel thiazole derivatives with antitubercular activity. Hydrazide–hydrazone **39** was found to be most active against *M. tuberculosis* H37Rv (MIC = 16.252 µg/mL) and had no cytotoxicity towards CRFK (Crandell Rees feline kidney) cells (CC_50_ > 100 µg/mL). However, its activity was lower than for rifampicin, which was used as a reference substance (MIC = 0.125 µg/mL) (Figure 31) [41].

**Figure 32:**
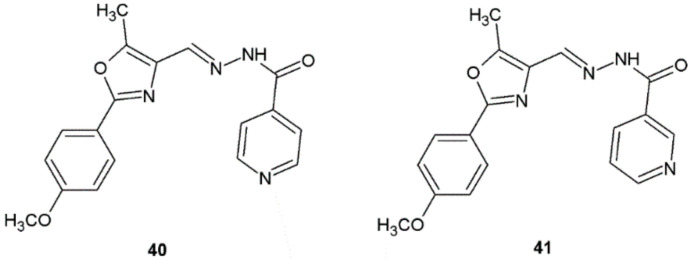
1,3-Oxazole-isoniazid hybrids with significant antitubercular activity.

New 1,3-oxazole-isoniazid hybrids synthesized by Shah et al. were evaluated for their antitubercular activity against *M. tuberculosis* H37Rv strain. Among all tested compounds, derivatives **40** and **41** displayed activity with MIC value of 1.56 μg/mL, which was higher when compared with ethambutol (MIC = 3.13 μg/mL). The authors discovered that compounds bearing methoxy groups in the phenyl ring attached to the 1,3-oxazole scaffold displayed better activity compared to the other compounds (Figure 32). Synthesized substances (**40**, **41**) were tested in vitro for cytotoxicity in human embryonic kidney (HEK-293T) cells and did not display changes in cytotoxicity as compared with vehicle (DMSO) [42].

### 2.3. Antifungal Activity

Treatment of fungal infections is very challenging even though we can use many medicines [43,44]. Due to this, searching for novel effective and non-toxic antifungal agents is necessary [43,44]. Many hydrazide–hydrazones beside antibacterial activity also possess interesting antifungal properties [14,15,16,17,18,19,21,23,26,29,45,46].

Krátký et al. evaluated hydrazide–hydrazones of 4-trifluoromethylbenzoic acid also as possible antifungal agents. The most active against a panel of fungi was, similarly as against bacterial strains, the hydrazide–hydrazone numbered as **4** (Figure 3, Table 13). The activity of this molecule (MIC = 1.98 µM) was four times higher than for fluconazole (MIC = 7.81 µM) against *Trichophyton mentagrophytes* 445 [14].

Compounds **5** and **6** synthesized by Abdelrahman et al. displayed antifungal activity (Figure 4). Their activity against tested fungi was higher or equal to the activity of Amphotericin B (Table 14) [15].

**Figure 33:**
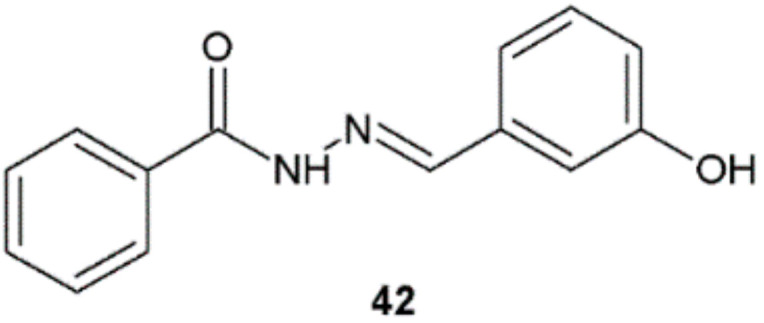
Hydrazide–hydrazone with antifungal activity.

Analysis of the diameter of zone of inhibition growth (mm) of compounds obtained by Manikandan et al. revealed that only hydrazide–hydrazone **42** had shown good antifungal sensitivity against three species of fungi (Figure 33, Table 15) [16].

**Figure 34:**
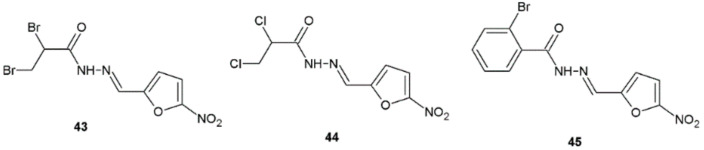
Nitrofurazone analogues with antifungal properties.

Nitrofurazone analogues synthesized by Popiołek and Biernasiuk also showed interesting antifungal activity. Compounds **43**, **44**, and **45** displayed good fungicidal or fungistatic properties against *Candida* spp. ATCC (MIC = 31.25–125 µg/mL, MFC = 125–1000 µg/mL) but they were less active than fluconazole, which was used as a reference substance (Figure 34) [17].

Benzimidazole derivatives synthesized by Yadav et al. were also tested for their antifungal activity. The best results showed compound **11** (Figure 7). Its activity against *C. albicans* (MIC = 0.016 µM) and *A. niger* (MIC = 0.032 µM) was higher than that of fluconazole (MIC = 0.40 and 0.82 µM, respectively) [18].

Molecules synthesized by El-Sayed et al. possessed interesting antifungal activity. Especially compounds **12** and **13** showed good effects against *Aspergillus niger* (diameter of zone of inhibition growth ZOI = 16 and 19 mm, respectively) in comparison with nystatin (ZOI = 20 mm) (Figure 8) [19].

All yeasts belonging to *Candida* spp. ATCC were sensitive to hydrazide–hydrazones of isonicotinic acid synthesized by Popiołek et al. Compound **15** had moderate fungicidal activity against *C. albicans* ATCC 2091 (MIC = 250 μg/mL, MFC/MIC = 4) and good fungistatic properties towards other *Candida* spp. (MIC = 62.5–125 μg/mL, MFC/MIC = 8–16) (Figure 10) [21].

**Figure 35:**
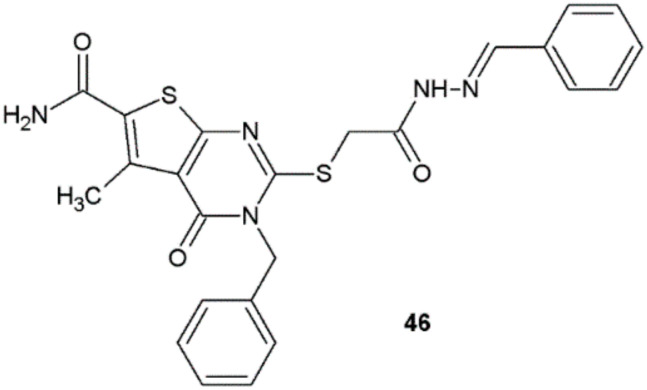
Pyrimidine derivative with antifungal properties.

Novel pyrimidine derivatives obtained by Shaaban et al. were tested in in vitro conditions for potential antifungal activity against *A. fumigatus*, *C. albicans*, and *Rhizopus oryzae*. The most active compound was **46**, which displayed half the activity (MIC = 25 μg/mL) of the reference clotrimazole (MIC = 12.5 μg/mL) against *C. albicans* and double the activity of clotrimazole against *A. fumigatus* with MIC = 50 μg/mL (Figure 35) [23].

**Figure 36:**
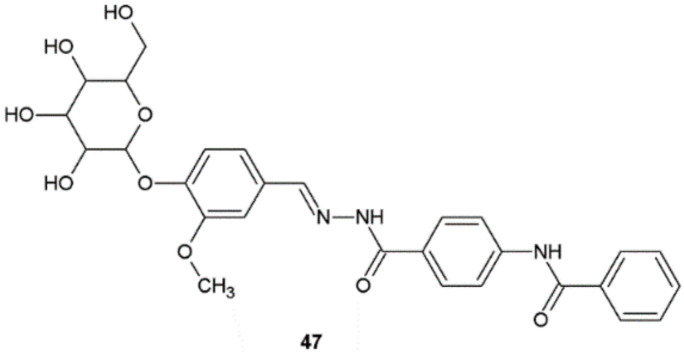
Hydrazide–hydrazone **47** with activity against *Candida* spp.

The antifungal activity of the new hydrazide–hydrazones synthesized by Guilherme et al. was evaluated against different strains of fungi. The compound **47** showed moderate antifungal activity (IC_50_ = 907.1 µM against *C. krusei* ATCC 6258 and IC_50_ = 226.8 µM against *C. parapsilosis* ATCC 22019) (Figure 36). This hydrazide–hydrazone displayed no cytotoxicity against kidney cell Vero line (ATCC CCL-81) CC_50_ > 181.4 µM and normal human lung fibroblast cell line MRC-5 (ATCC CCL-117) CC_50_ = 104.3 ± 2.4 µM [45].

Among novel indol-2-one derivatives with hydrazide–hydrazone moiety compound, **21** was found to have the most potent antifungal properties, even though its activity against *C. albicans* (MIC = 31.25 µg/mL) was two times and for *F. oxysporum* (MIC = 125 µg/mL) four times lower than for amphotericin B (MIC = 15.62 and 31.25 µg/mL, respectively) (Figure 15) [26].

New hydrazide–hydrazones of 5-nitrofuran-2-carboxylic acid obtained by Popiołek et al. also indicated significant anticandidal activity. The values of MIC of compound **26**, which contained the 2-iodophenyl substituent, were the lowest (from 7.81 to 15.62 μg/mL), indicating its strong or very strong activity against all reference *Candida* spp. (Figure 18) [29].

**Figure 37:**
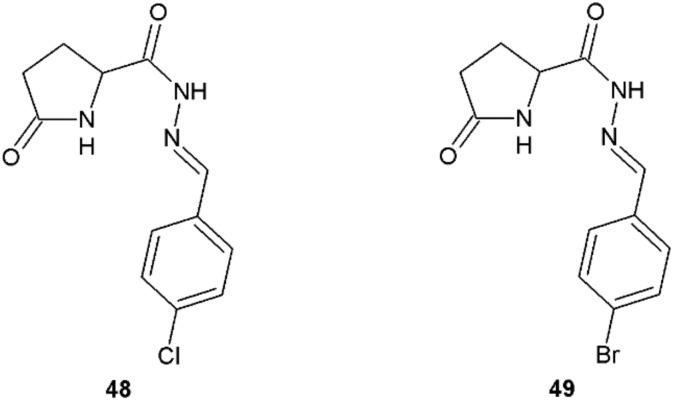
5-Pyrrolidin-2-one derivatives **48** and **49** with antifungal properties.

Among novel derivatives of 5-pyrrolidin-2-one, synthesized by Dascalu et al., compounds **48** and **49** with chloride and bromine atom at position 4 in the phenyl ring showed a broad spectrum of antifungal activity (Figure 37, Table 16) [46].

## 3. Conclusions

In conclusion, this article gives an overview of the antibacterial, antitubercular, and antifungal properties of hydrazide–hydrazones published since 2017. As presented in this study, the hydrazide–hydrazone moiety may be found and incorporated in various bioactive molecules. Thus, this review appears to be important for further development of hydrazide–hydrazones as potential antimicrobial agents.

## Figures and Tables

**Table 1:** The results of minimal inhibitory concentration (MIC) in µg/mL of compound **3**.

Compound	Minimal Inhibitory Concentration (MIC) (µg/mL)					
	*P. aeruginosa*	*S. aureus*	*E. coli*	*B. licheniformis*	*P. vulgaris*	*M. varians*
**3**	1.56 ± 0.02	0.39 ± 0.02	0.78 ± 0.02	1.56 ± 0.02	0.39 ± 0.02	0.78 ± 0.02

**Table 2:** The antibacterial activity values of compound **4**.

Compound	Minimal Inhibitory Concentration (MIC) [µM]									
	SA		MRSA		SE		EF		EC	
	24 h	48 h	24 h	48 h	24 h	48 h	24 h	48 h	24 h	48 h
**4**	1.98	1.98	1.98	1.98	3.9	3.9	1.98	3.9	250	250
BAC	7.81	15.62	15.62	15.62	15.62	31.25	15.62	62.5	>500	>500

BAC—bacitracin; Bacteria: SA—*Staphylococcus aureus* CCM 4516/08; MRSA—methicillin-resistant *Staphylococcus aureus* H 5996/08; SE—*Staphylococcus epidermidis* H 6966/08; EF—*Enterococcus faecalis* J 14365/08; EC—*Escherichia coli* CCM 4517.

**Table 3:** Antibacterial activities of *N*-(4-fluorobenzylidene)benzohydrazide.

Compound	Diameter (mm) of Zone of Inhibition Growth							
	SA	ML	BS	KP	VP	PM	EC	PA
**7**	6	8	6	6	8	6	7	6
Ciprofloxacin	10	9	12	9	8	10	9	8

SA—*Staphylococcus aureus*; ML—*Micrococcus luteus*; BS—*Bacillus subtilis*; KP—*Klebsiella pneumoniae*; VP—*Vibrio parahaemolyticus*; PM—*Proteus mirabilis*; EC—*Escherichia coli*; PA—*Pseudomonas aeruginosa*.

**Table 4:** Antibacterial activity of compounds **12** and **13**.

Compound	Diameter (mm) of Zone of Inhibition Growth			
	Gram-Positive Bacteria		Gram-Negative Bacteria	
	*B. subtilis*	*B. cereus*	*P. aeruginosa*	*E. coli*
**12**	33	15	16	21
**13**	46	18	22	19
Cefotaxime	32	28	32	34

**Table 5:** Antibacterial properties of compound **17**.

Compound	MIC (µmol/mL)	Microorganism			
		*S. aureus* ATCC 6538	*E. coli* ATCC 10536	MRSA MFBF 10679	ESBL+ *E. coli* MFBF 12794
**17**	IC_90_ IC_50_	0.12 4.7 × 10^−2^	0.37 0.35	2.30 × 10^−2^ 2.10 × 10^−2^	0.36 0.16
Gentamicin Sulphate	IC_90_ IC_50_	1.35 × 10^−4^ 5.03 × 10^−5^	>3.35 × 10^−2^ >3.35 × 10^−2^	6.97 × 10^−4^ 2.97 × 10^−4^	>3.35 × 10^−2^ >3.35 × 10^−2^
Norfloxacin	IC_90_ IC_50_	>6.26 × 10^−3^ >6.26 × 10^−3^	4.95 × 10^−5^ 4.68 × 10^−5^	>6.26 × 10^−3^ >6.26 × 10^−3^	>6.26 × 10^−3^ >6.26 × 10^−3^
Colistin	IC_90_ IC_50_	>2.16 × 10^−2^ >2.16 × 10^−2^	>2.16 × 10^−2^ >2.16 × 10^−2^	>2.16 × 10^−2^ >2.16 × 10^−2^	5.07 × 10^−5^ 4.63 × 10^−5^

ESBL+ *E. coli*—extended-spectrum beta-lactamase-positive *E. coli.*

**Table 6:** Minimal inhibitory concentration (MIC) and minimal bactericidal concentration (MBC) of compound **18**.

Compound	Minimal Inhibitory Concentration (MIC) and Minimal Bactericidal Concentration (MBC) in µg/mL					
	Gram-Positive Bacteria			Gram-Negative Bacteria		
	*S. aureus*	*S. epidermidis*	*B. subtilis*	*P. aeruginosa*	*E. coli*	*P. vulgaris*
	MIC MBC	MIC MBC	MIC MBC	MIC MBC	MIC MBC	MIC MBC
**18**	50 50	50 100	25 50	25 50	50 50	100 100
Ampicillin	6.25	12.5	12.5	-	-	-
Levofloxacin	-	-	-	12.5	6.25	12.5

**Table 7:** Minimal inhibitory concentration (MIC) of hydrazide–hydrazone **20**.

Compound	MIC (µM)		
	Gram-Positive Bacteria		
	*E. faecalis* ATCC 13124	*S. aureus* ATCC 25923	*B. cereus* ATCC 13245
**20**	12.5	12.5	12.5
Streptomycin	350	350	175

**Table 8:** In vitro antimicrobial activity values (MIC, µg/mL) for compound **21**.

Compound	Minimal Inhibitory Concentration (MIC) (µg/mL)			
	Gram-Positive Bacteria		Gram-Negative Bacteria	
	*B. subtilis*	*S. aureus*	*E. coli*	*P. aeruginosa*
**21**	15.62	55.5	7.81	83.33
Tetracycline	31.25	62.5	15.62	62.5

**Table 9:** The IC_50_ values for compound **22**.

Compound	IC_50_ (μM)		
	*E. coli* MTCC 433	*P. putida* MTCC 1237	*B. subtilis* MTCC 1427
**22**	0.19 ± 0.06	0.45 ± 0.18	0.14 ± 0.02
Chloramphenicol	0.23 ± 0.05	0.25 ± 0.02	0.21 ± 0.06

**Table 10:** Diameter of zone of inhibition growth for substance **23**.

Compound	Diameter of Zone of Inhibition Growth (mm/mg Sample)				
	Gram-Positive Bacteria		Gram-Negative Bacteria		
	*B. cereus* ATCC 11778	*S. aureus* ATCC 29213	*S. typhimurium* ATCC 14028	*E. coli* ATCC 25922	*P. aeruginosa* ATCC 27953
**23**	10	30	30	10	30
Penicillin	25	32	40	15	-
Nizo-arm	-	-	-	-	45

**Table 11:** Antibacterial properties of compound **27**.

Compound	Diameter (mm) of Zone of Inhibition Growth at 50 µg/mL		
	Gram-Negative Bacteria		Gram-Positive Bacteria
	*E. coli*	*P. aeruginosa*	*S. aureus*
**27**	40	28	25
Ciprofloxacin	40	50	35

**Table 12:** Antitubercular properties of compound **29**.

Compound	MIC [µM]									
	*M. tuberculosis* 331/88		*M. avium* 330/88		*M. kansasii* 235/80			*M. kansasii* 6509/96		
	14 day	21 day	14 day	21 day	7 day	14 day	21 day	7 day	14 day	21 day
**29**	16	16	>125	>125	16	16	16	16	16	16
Isoniazid	0.5	1	>250	>250	>250	>250	>250	8	8	8

**Table 13:** Antifungal properties of compound **4**.

Compound	MIC [µM]							
	CT		CK		CG		TM	
	24 h	48 h	24 h	48 h	24 h	48 h	24 h	48 h
**4**	>125	>125	>125	>125	≤0.49	0.98	1.98	3.9
Fluconazole	>500	>500	125	250	31.25	500	7.81	125

Fungi: CT—*Candida tropicalis* 156; CK—*Candida krusei* E28; CG—*Candida glabrata* 20/I; TM—*Trichophyton mentagrophytes* 445.

**Table 14:** Antifungal activity displayed as MIC values (µg/mL) of tested standard and compounds **5** and **6**.

Compound	MIC (µg/mL)	
	*A. fumigatus* RCMB 02568	*C. albicans* RCMB 05036
**5**	0.98	0.49
**6**	0.98	0.98
Amphotericin B	1.95	0.98

**Table 15:** Antifungal activity of *N*-(3-hydroxybenzylidene)benzohydrazide.

Compound	Diameter of Zone of Inhibition Growth (mm)		
	*A. niger*	*Mucor* spp.	*Trichoderma viride*
**42**	6	6	7
Miconazole	7	8	10

**Table 16:** The MIC_50_ values of compounds **48** and **49** against fungal strains.

Compound	MIC_50_ (µg/mL)				
	*Fusarium solani*	*Penicillium ochrochloron*	*Cladosporium cladosporioides*	*Geotrichum candidum*	*Candida tropicalis*
**48**	-	15.4	23.9	1.8	-
**49**	6.5	5.3	53.0	5.9	>75
Ketoconazole	-	-	-	1.5	15.9
Hymexazol	15.6	62.2	28.9	>50	-
Fluconazole	-	-	-	1.6	-
